# Supplementary material for: Getting the nod: Pediatric head motion in a transdiagnostic sample during movie- and resting-state fMRI
Source: PLoS One. 2022 Apr 14;17(4):e0265112. doi: 10.1371/journal.pone.0265112 (PMC9009630; doi:10.1371/journal.pone.0265112)
Supplement: S1 Fig — The HBN database uses a community-based recruitment strategy that encourages enrollment of participants with behavioural or psychiatric concerns, as well as typically developing children and youth. Here we show distributions of three measures relevant to in-scanner head motion: IQ, anxiety, and ADHD. These distributions indicate that the sample is psychiatrically enriched, but also that the full spectrum of each measure is represented (i.e., that the extreme scores are not dominating the sample) (Alexander et al., 2017). Red dashed lines indicate the cut-off or threshold for clinical concern. (PDF) [file pone.0265112.s001.pdf]

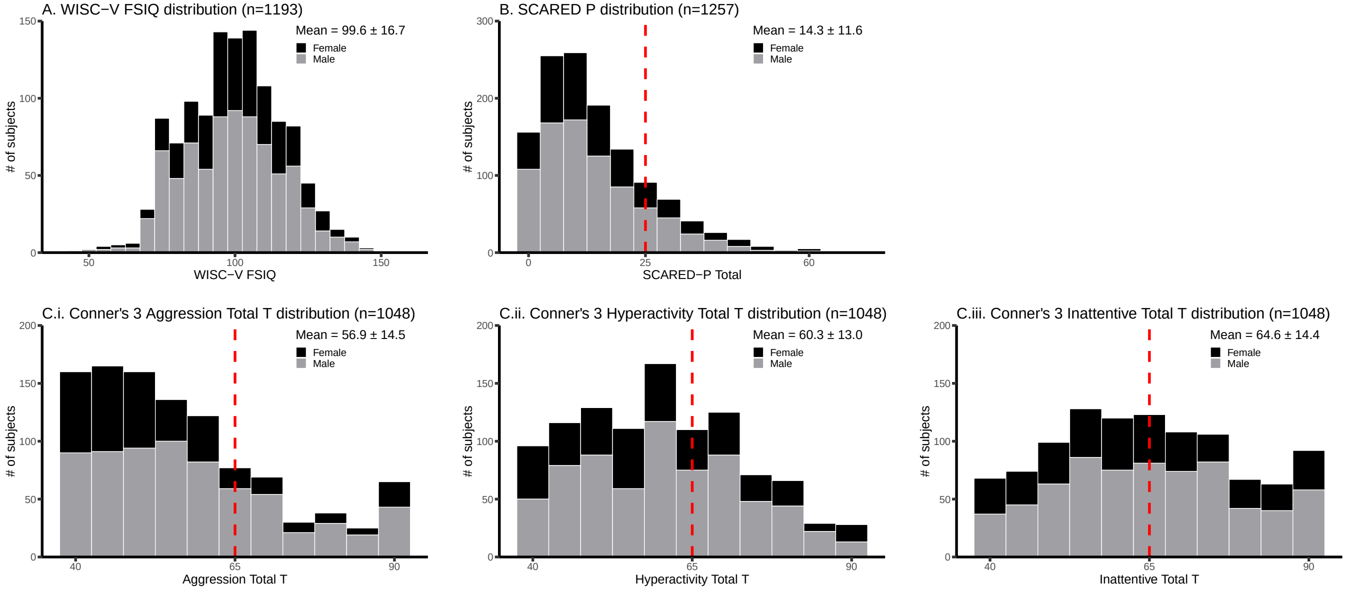

Fig S1. Additional sample demographics of clinical measures. The HBN database uses a community-based recruitment strategy that encourages enrollment of participants with behavioural or psychiatric concerns, as well as typically developing children and youth. Here we show distributions of three measures relevant to in-scanner head motion: IQ, anxiety, and ADHD. These distributions indicate that the sample is psychiatrically enriched, but also that the full spectrum of each measure is represented (i.e., that the extreme scores are not dominating the sample) (Alexander et al., 2017). Red dashed lines indicate the cut-off or threshold for clinical concern.
