# Supplementary material for: Getting the nod: Pediatric head motion in a transdiagnostic sample during movie- and resting-state fMRI
Source: PLoS One. 2022 Apr 14;17(4):e0265112. doi: 10.1371/journal.pone.0265112 (PMC9009630; doi:10.1371/journal.pone.0265112)
Supplement: S2 Fig — All participants were binned by age, and mean framewise displacement is shown for Movie and Rest1 for each bin. We again show that the advantage of lower mean FD with movies applies below age 11 years (Vanderwal 2018, Greene 2018). Qualitatively, motion amplitude and patterns look similar from ages 13–21 years, indicating that at around age 13, head motion at the group level looks adult-like. (PDF) [file pone.0265112.s002.pdf]

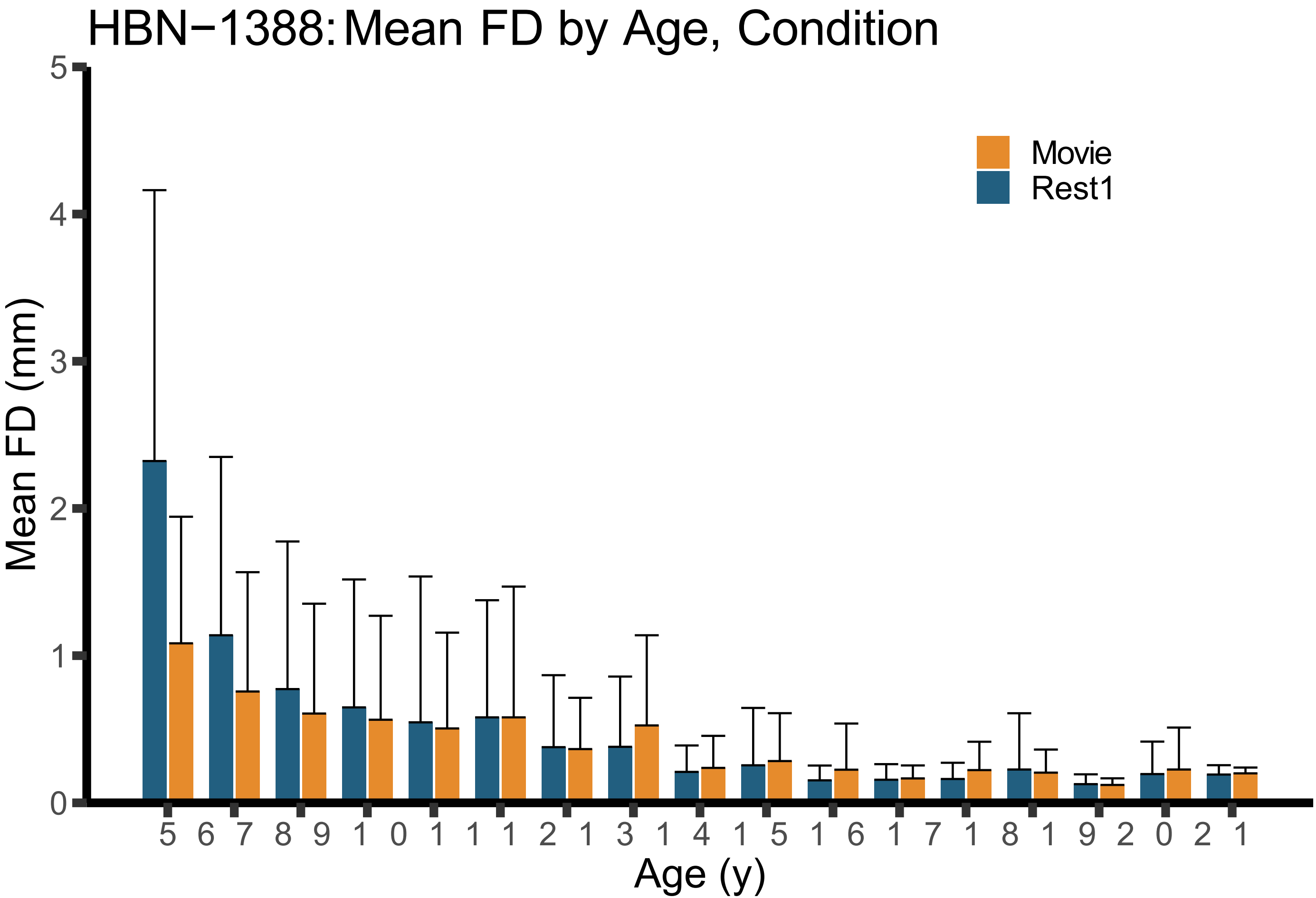

Fig S2. Mean FD by age and condition (HBN-1388). All participants were binned by age, and mean framewise displacement is shown for Movie and Rest1 for each bin. We again show that the advantage of lower mean FD with movies applies below age 11 years (Vanderwal 2018, Greene 2018). Qualitatively, motion amplitude and patterns look similar from ages 13-21 years, indicating that at around age 13, head motion at the group level looks adult-like.
